# An Endovascular Catheterization Robotic System Using Collaborative Operation with Magnetically Controlled Haptic Force Feedback

**DOI:** 10.3390/mi13040505

**Published:** 2022-03-25

**Authors:** Xinming Li, Shuxiang Guo, Peng Shi, Xiaoliang Jin, Masahiko Kawanishi

**Affiliations:** 1 Graduate School of Engineering, Kagawa University, Takamatsu 761-0396, Japan; s21d504@kagawa-u.ac.jp (X.L.); s19d503@stu.kagawa-u.ac.jp (P.S.); s19d505@stu.kagawa-u.ac.jp (X.J.); 2 Key Laboratory of Convergence Medical Engineering System and Healthcare Technology, Ministry of Industry and Information Technology, School of Life Science and Technology, Beijing Institute of Technology, Beijing 100081, China; 3 Department of Neurological Surgery, Kagawa University, Takamatsu 761-0793, Japan; mk@med.kagawa-u.ac.jp

**Keywords:** endovascular catheterization robotic system (ECRS), magnetically controlled haptic (MCH) force feedback, collaborative operation, robot-assisted surgery

## Abstract

Robot-assisted technology is often used to perform endovascular catheterization surgeries, which generally depend on the flexible operability and the accurate force feedback of a robotic system. In this paper, an endovascular catheterization robotic system (ECRS) was developed to improve collaborative operation and haptic force feedback. A couple of operating handles were designed to maximize the use of the natural operations of surgeons on the master side, which is a flexible and ergonomic device. A magnetically controlled haptic force feedback structure is proposed based on hydrogel and solid magnetorheological (MR) fluid to offer a sense of haptic feedback to operators; this has potential influence on the field of force feedback. In addition, a unique tremor-reduction structure is introduced to enhance operating safety. Tracking performance experiments and in vitro experiments were conducted to evaluate the performance of the developed ECRS. According to these experimental results, the average translation-tracking error is 0.94 mm, and the average error of rotation is 0.89 degrees. Moreover, in vitro experiments demonstrated that haptic feedback has the advantage of reducing workload and shortening surgery completion time. The developed ECRS also has the benefits of inspiring other researchers to study collaborative robots and magnetically controlled feedback.

## 1. Introduction

**Figure 1:**
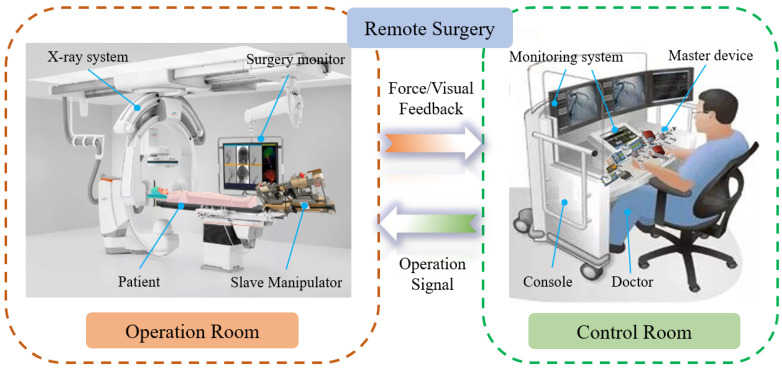
Schematic of the robot-assisted endovascular catheterization remote surgery.

Robot-assisted endovascular catheterization (EC) systems are attracting growing interest and attention due to the obvious drawbacks of traditional vascular interventional surgery (VIS), including prolonged X-ray radiation, high-intensity surgical stress and unmonitored surgical risks [1]. In the field of robot-assisted VIS, the core components are surgeons, patients, and robot operating systems [2,3]. As shown in Figure 1, endovascular interventional doctors could be liberated from the operating room in X-ray radiation, and their long-term workload reduced. Surgeons operate a master device to control and monitor the overall process of interventional surgery in a separate control room without radiation. Correspondingly, the patient and slave manipulator are located in operating room for the performance of EC surgeries including insertion, extraction, and rotation. Robot-assisted surgeries also play a critical role in increasing the treatment comfort of patients and improving surgical precision. Furthermore, the design of master–slave makes it possible to develop the technology of telesurgery and specialist treatment during the process of VIS, which is of great significance for medical treatment in remote areas [4].

Recently, robot-assisted EC surgeries, including the structure of the master–slave system, haptic force feedback, and tremor reduction, have been studied in a few major fields [5]. Flexible and highly accurate master–slave design is vital to increase transparency for surgeons and slave robots, which directly affects the success of surgery. Haptic force feedback is another indispensable part of EC systems that reproduces the force information of surgical instruments for operators when conducting surgery. Surgeons on the master side can feel the sensation of haptic feedback and perform the procedure more safely. In addition, the design of tremor reduction would reduce operating errors on the master side and, thus, improve the surgical accuracy of the overall robotic system.

The current related efforts of robot-assisted EC systems can be divided into two categories: the design of the robotic system and the guarantee of safe operation. For studies about the design of the robotic system, an incipient EC robot was proposed to deal with the problem of remote surgery based on the “Slave–Master” structure. Additionally, a smart material, MR (magnetorheological) fluid, was proposed as the leading solution to providing flexibly controlled and effective haptic force feedback [6]. In [7], the operation performance and dynamic kinematics for a VIS robot were improved using a haptic feedback strategy and in vitro experiments, but the master device in this system is not flexible enough. A remote robot-assisted surgical system was proposed to improve operating precision based on a commercial phantom-and-slave insertion manipulator, which was an assembled operation device with considerable freedom [8]. Particularly, this kind of EC system has successfully conducted human body experiments, which were reported by news at the time. Moreover, this team also developed an isomorphic interactive device to duplicate the surgical skills of doctors, and which can adapt to complex operating environment for remote surgeries [9]. Kundrat D. et al. used an MR-safe teleoperation robotic system to enhance surgical performance, with a successful operation rate of 90–100% [10,11]. However, this system suffered from an inability to achieve collaborative operation. As for the efforts to guarantee of safe operation, Kit-Hang L. et al. introduced a robotic manipulator to offer high-resolution images to visualize lesions in a magnetic resonance imaging (MRI) environment; however, the authors made no mention of force feedback [12]. A new catheterization system was designed for accuracy research purposes with an accuracy higher than 90% in clinical applications [13]. However, the ergonomic design was still immature.

In addition, an augmented endovascular robotic system using conventional surgical tools was created to generate feedback and ensure operation safety [14], and a two-dimensional fuzzy PID controller was also equipped for a VIS robot [15]. Wang K. et al. developed a 12-DOF (degrees of freedom) robot with four manipulators to accurately clamp; rotation; and a push–pull guide wire and catheters [16,17]. However, the large size is a major drawback that cannot be ignored. Based on these studies and surgeons’ natural operation habits, EC robots are still lacking an ideal ergonomic design for the master device.

Focusing on the research of haptic force feedback, Lai W. et al. designed a tendon-sheath force sensor to measure the compression force with error of 0.178 N and sensitivity of 34.14 pm/N [18], but its applicability for EC robots was not determined. A small and highly sensitive visual force senor was presented to collect the acquired signal and force [19]. Despite this sensor’s ability to provide more intuitive force feedback for doctors, it was not a suitable product to replicate force. Additionally, Wei F. et al. proposed a unified framework for human haptic perception to address time delays in teleoperation [20]. According to recent efforts and the latest technologies for EC robots [21,22,23], vison-based detection [24,25], a hydraulically steerable guidewire/catheter [26,27], morphological and positional analysis [28], and magnetically driven navigation [29,30,31,32] are catering to a tremendous boom in the improvement of physicians’ surgeries and the enhancement of the patient experience. Nevertheless, the implementation of high-accuracy force feedback is still a challengeable area of study for robot-assisted EC systems.

Thus far, numerous commercial robotic systems, such as the Corpath^®^ robot, Magellan^TM^ robotic system, and Amigo^TM^ surgical robot, have been developed to assist with VIS in hospitals. Unfortunately, these commercial robots cannot realize force feedback or achieve precise haptic force feedback.

With the aims of safe robot-assisted surgery, high-precision force feedback, and ergonomic design for robot-assisted surgeries, An endovascular catheterization robotic system (ECRS) using collaborative operation with magnetically controlled haptic force feedback is developed in this paper. This system makes it possible to follow doctors’ natural operating habits and provides haptic feedback when conducting surgeries; it has the potential application value of reducing workload, improving the surgical environment, shortening surgery time, and enhancing operating safety for surgeons. Compared with other robotic systems in the field of VIS, our ECRS has two innovative advantages in manipulating robot-assisted surgery. Firstly, the collaborative operation master device based on a couple of handles enables maximum replication of the physician’s operation. Secondly, we proposed the magnetically controlled haptic force feedback molded by solid MR fluid and the smart material hydrogel, which is a new case in cross-disciplinary application. This also has potential application value in the fields of haptic feedback, controlled clamping, and biological self-healing.

The remainder of this article is organized as follows: Section 2 introduces the design and control architecture of the developed ECRS; the principles and dynamic analysis of the robotic system are described in Section 3; an experimental performance evaluation of the ECRS is presented in Section 4; and lastly, Section 5 states the conclusion of this paper.

## 2. Design and Control Mechanism of the Developed ECRS

This section presents the mechatronics of our developed ECRS. The ECRS is a teleoperated surgical system. It is made up of a master haptic device, a slave manipulator-robot, and control mechanism. A detailed description of the master haptic device, slave manipulator-robot, and system control architecture are given in this section. The master device is mounted in the control room (master side) to be operated by surgeons. Correspondingly, the slave manipulator is installed on the operating bed or independent robotic arm (slave side) to deliver surgical instruments and detect necessary force and position information. The system control mechanism is used to exchange information between the master device and slave manipulator.

### 2.1. Master Haptic Device

**Figure 2:**
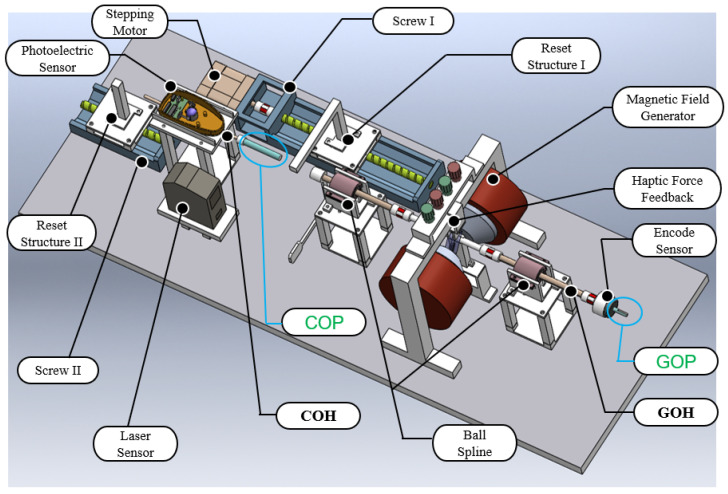
The designed master haptic device. Detailed components of master side: COP (catheter operating position); COH (catheter operating handle); GOP (guidewire operating position); GOH (guidewire operating handle); reset structure II (motor-driven part is omitted).

In the developed ECRS, a master haptic device is designed as a collaborative operation structure to control the corradiated movement of the catheter and guidewire on the slave side. The fundamental purpose of the master device, which is operated by surgeons, is to capture the axial and radial motions of the guidewire and catheter. Notably, the haptic force feedback of the master side is a significant research issue, and is used for providing operators with the contact force status between medical instrument and blood vessel of the patient when conducting an interventional surgery. Figure 2 shows the proposed master haptic device. Two independently designed handles can achieve a collaborative operation with doctors. The details of our designed master haptic device will be introduced as follows:

(1) ***Guidewire operating handle (GOH):*** In order to capture the radial motion of the input guidewire, an encode sensor (MTL, MES020-2000p, Japan) is mounted to the right of the GOH. A laser sensor (KEYENCE, LK-2500) installed in this handle has the ability to detect the real-time axial motion. At the same time, the structure of the haptic force feedback will provide specific resistance for operators when manipulating the input GOH. A detailed introduction of haptic force feedback will be presented in Section 3.1. Reset structure I is located on the screw I, and is driven by a stepping motor (ASM46AA, ORIENTA MOTOR, Japan). The resetting design makes it is possible to reproduce the repeated push–pull action. In addition, two ball splines (THK, SLT 006-T2-N5) are installed to support and smooth the axial motion of the GOH. The guidewire operating position (GOP) for doctors lies to the right of encoder (See GOP in Figure 2).
(2) ***Catheter operating handle (COH):*** With the aim of collecting the motion information of the input catheter, we utilize a high-level integrated photoelectric sensor (PS). This kind of PS includes two photoelectric measuring cells, which are perpendicular to each other. The movement of the handle rod drives the trackball into rotation, then two photoelectric measuring cells detect the motion signal. The biggest advantage of employing the PS is that it can simultaneously capture translational motion and rotational motion, which will greatly shorten the length of operating handle. Meanwhile, reset structure II, mounted on the screw II, is also able to cut down the distance of the COH. Lastly, the catheter operating position (COP) for surgeons is marked with a blue circle (See COP in Figure 2). Moreover, the proposed master haptic device can be collaboratively operated using two handles with different mechanics and sensors. The related technical parameters of the designed master haptic device are shown in Table 1.

### 2.2. Slave Manipulator-Robot

**Figure 3:**
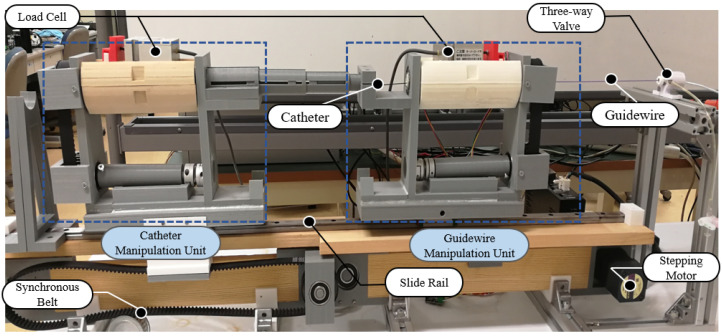
The slave manipulator-robot. Detailed components of slave side: CMU (catheter manipulation unit), GMU (guidewire manipulation unit), Surgical instruments (catheter and guidewire). For more details, see [3].

The surgical instruments, such as the catheter and guidewire, are inserted into the blood vessels of patients by a slave manipulator-robot, which is placed in the operating room. This slave manipulator was designed by our research team in a previous study [3], and is shown in Figure 3. This slave side has the ability to collaboratively operate the guidewire and catheter simultaneously, thanks to the designs of the catheter manipulation unit (CMU) and guidewire manipulation unit (GMU). The insertion motion and extraction motion of CMU and GMU are achieved using a stepping motor (ASM46AA, ORIENTAL MOTOR, Japan) with a resolution of 0.36 degrees. Meanwhile, two torque sensors are used to collect several radial torque signals of the catheter and guidewire. It should be noted that a novel grasp device based on two micro-stepping motors (LIKO MOTOR, 20BYGH30-0604A) was adopted on the slave side. In addition, the proximal force of the moving instrument is measured by two load-cells (TU-UJ, TEAC, Japan) with a detection range from −5 N to 5 N, and the force value is exhibited in the control console. The maximum effective moving range of CMU and GMU is 40 cm, and the overall length of this slave manipulator-robot is 92 cm. This slave manipulator-robot can insert/extract a surgical guidewire and catheter with high precision and effectively detect force when conducting an endovascular catheterization surgery.

### 2.3. System Control Architecture

**Figure 4:**
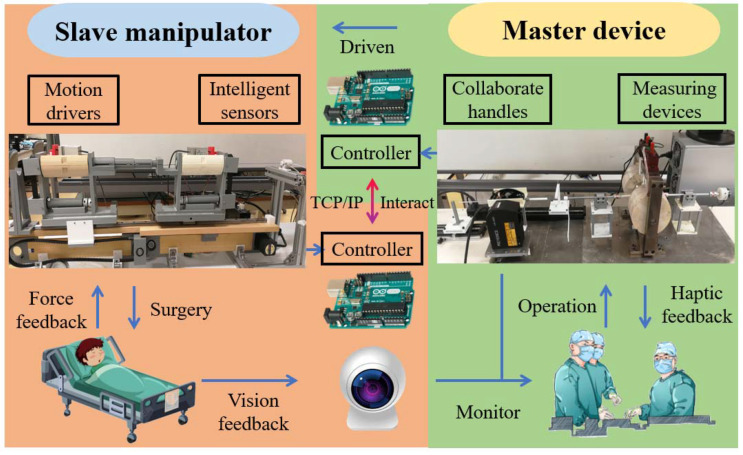
The control architecture diagram of the developed ECRS.

The control mechanism of the developed master–slave ECRS mainly includes the slave manipulator, master device, and interactive communication. The core controller used in this ECRS is the AT Mega 2560 (Arduino, Turin, Italy) control board, which can capture operations from the master side and drive the movements of the guidewire and catheter on the slave side. Additionally, the force signal and position signal of the slave side can be detected in real-time, and the vision feedback is provided by a network camera (VIV-TEK, USA). On the master side, the monitor information is displayed on a screen, which can guide the doctor’s treatment process. The system control architecture developed for the ECRS is shown in Figure 4.

## 3. Principles and Dynamics of the System

Considering the core research challenges of EC robots, the haptic feedback on the master side and the force detection mechanism on the slave side will be presented in this section. The purpose of haptic force feedback is to improve the robotic environment by providing a surgical-resistance sensation to operators. Meanwhile, the collaborative operation process is introduced for surgeons when conducting surgery using the proposed master haptic device.

### 3.1. Implementation of Haptic Force Feedback

In order to realize the haptic force feedback on the master side, we propose a magnetically controlled haptic force feedback structure based on a solid magnetorheological (MR) material and hydrogel rubber. This section describes the details of the implementation of haptic force feedback in our study. The novel implementation of this method consists of component preparation, a molding process, and structure assembly, which are introduced as below.

**Figure 5:**
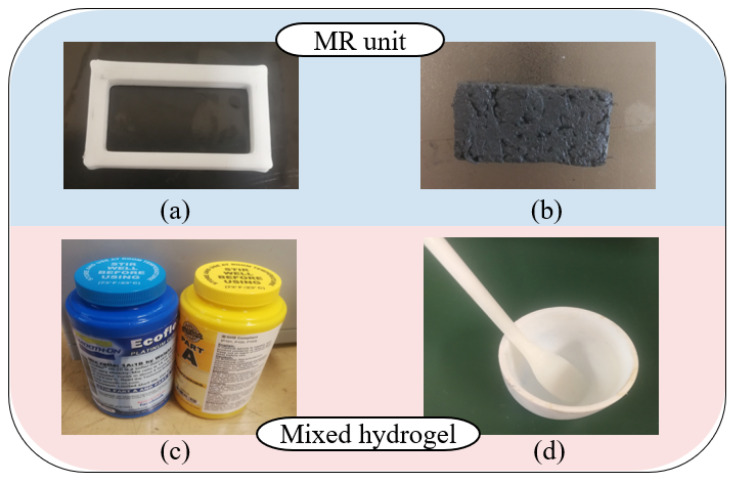
The related components for preparation: (**a**) 3D-printed mold used for MR unit; (**b**) actual display of MR unit; (**c**) mixture A and mixture B for hydrogel rubber; (**d**) porcelain container for mixing.

**Figure 6:**
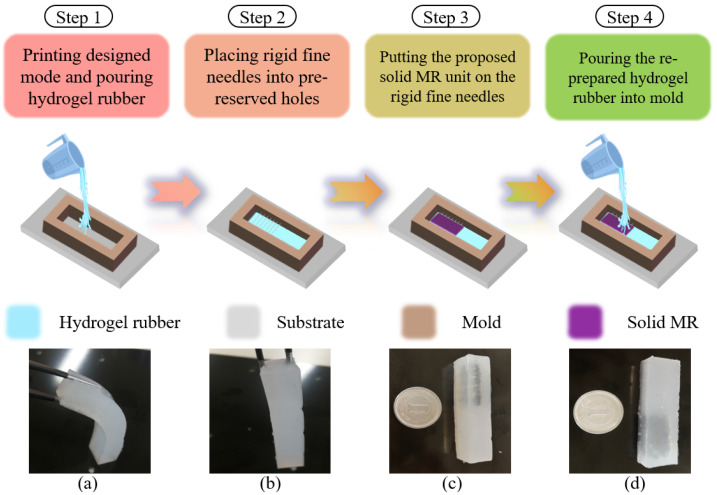
The molding process for magnetically controlled hydrogel (MCH): (**a**) bendability of hydrogel without MR unit; (**b**) self-healing of hydrogel without MR unit; (**c**) the side view of MCH; (**d**) the main view of MCH.

**Figure 7:**
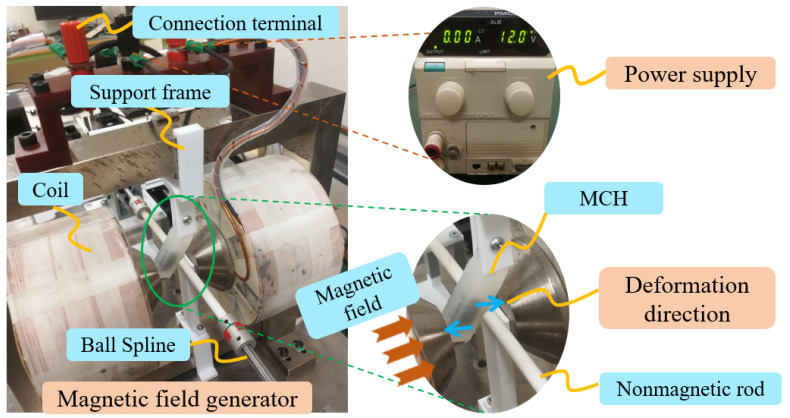
The magnetically controlled haptic force feedback based on MCH and the way to provide force feedback.

(1) ***Component preparation:*** Earlier research by our team adopted MR fluid to generate haptic force feedback [6,7]. This kind of smart material displays good controllability under a magnetic field. Since it is a substance in which oil and liquid are layered, we selectively use the liquid part for this study. Then, a firming agent is added to improve the plasticity of the MR material. After forming in a rectangular mold, a solid MR unit is obtained with a specific shape. In addition, the hydrogel rubber (Ecoflex^TM^, 00-20, Macungie, PA, USA) includes mixture A and mixture B, which need to wait for their pot-life of 30 min. The mixed liquid should also be prepared before the molding process. Figure 5 shows some related components.
(2) ***Molding process:*** After the process of preparation, four steps are proposed to build the magnetically controlled hydrogel (MCH), which will be employed to offer haptic feedback. The detailed molding process and generated samples are shown in Figure 6.
(3) ***Structure assembly:*** This part is the last procedure to build the complete haptic force feedback structure. It is important to assemble the MCH with 3D-printed parts, which should be mounted under a magnetic environment. Figure 7 shows an actual view of the haptic force feedback structure and the way in which it provides force feedback. An adjustable power supply is used to directly offer current to the magnetic field generator. When supplying current to the magnetic field generator, two coils will generate magnetic fields to control the deformation of the MCH. The magnetic density is detected using a Tesla Meter (KANETEC, Japan).

The underlying principle of “haptic device” is frictional resistance generated by contact between the MCH and a nonmagnetic rigid rod. As shown in Figure 7, the upper and lower parts of the MCH, placed at an inclined angle, incur relative deformations that will result in direct contact between the MCH and the nonmagnetic rod. With the change in magnetic field density, the MCH can undergo various degrees of deformation to provide different force feedback sensations to the operator’s hand. In other words, we use the current to control the magnetic field density, and adopt a magnetic field to control the force feedback sensation generated by the MCH.

### 3.2. Structural Design of Reducing Operating Tremors

**Figure 8:**
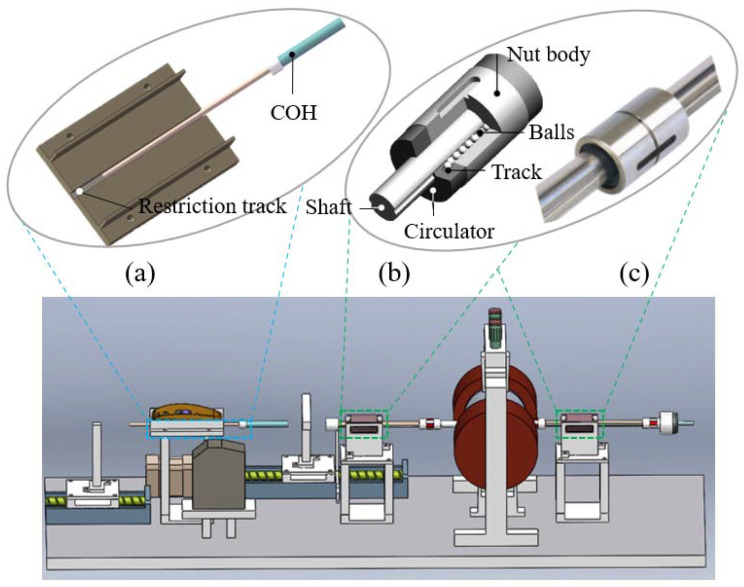
Structural design of operating tremor reduction in the master haptic device: (**a**) restriction track for COH; (**b**) structure diagram of ball spline; (**c**) actual picture of ball spline.

In our proposed master haptic device, the reduction in operational tremors is a highlight that cannot be ignored. The appearance of hand tremors in VIS will directly affect the accuracy of the translation motion and the rotation motion of the master–slave robot. Hence, we designed a related structure to reduce operating tremors. We designed a restriction track with a length of 10 cm to deal with unknown tremors, which are generated in catheter operating handle (COH). In addition, the guidewire operating handle (GOH) adopts two ball splines (THK, SLT 006-T2-N5) to suppress tremors of the hand, and also has the function of supporting the COH. Ball splines are famed for their small resistance and smooth running, based on the special structure of the recirculating balls and track, which are shown in Figure 8.

### 3.3. Analysis of the Collaborative Operation Mechanism on Master Side

**Figure 9:**
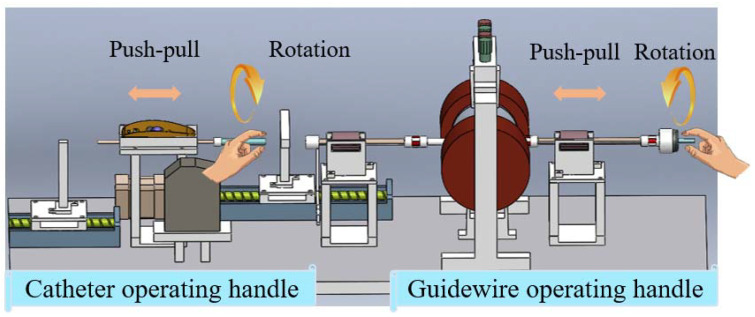
The collaborative operation mechanism of the designed master haptic device.

Collaborative operating is vital for surgeons when conducting interventional surgeries. In this study, the coaxial design of the handles and the separate structure of the catheter handle and the guidewire handle enable collaborative operations to be performed in accordance with the surgeon’s natural surgical method. The collaborative operation mechanism is exhibited in Figure 9. Both the COH and GOH have a translation motion (push–pull) and a rotation motion. By selectively manipulating the COH and GOH, the status of the translation or rotation can be determines, and individual movement, individual rotation, synchronized movement, and synchronized rotation can be accomplished in operations.

### 3.4. Analysis of the Force Detection Model on Slave Side

**Figure 10:**
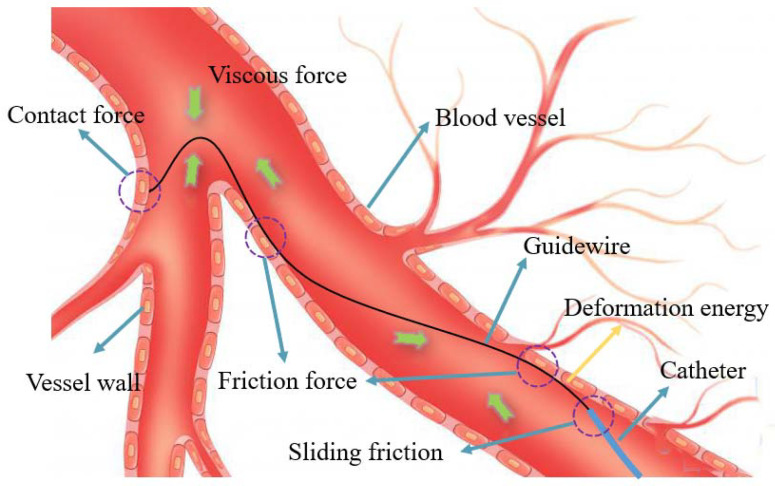
Detection force analysis of the catheter and guidewire.

Force detection on the slave side is an important issue, and can capture real-time force information during surgery. This detected force can also be used for haptic feedback of the master side. Two load-cells mounted on the slave manipulator-robot detect the proximal force in the developed ECRS. As shown in Figure 10, five force components make up the complete proximal force: viscous force, contact force, sliding friction, friction force, and potential deformation energy. The viscous force is the natural viscous resistance from blood, which is inevitable. The contact force is a collision force between the tip of guidewire and the blood vessel wall. Unlike the instantaneity of the contact force, the friction force is a combined force with lots of curved contact points that occur over the whole surgical instrument. Sliding friction is also a kind of friction force, and defines the interaction force between the inner wall of catheter and the outer wall of guidewire. Finally, the vital potential deformation energy mentioned in this section is caused by the irregular deformation of an elastic object, such as a surgical instrument. Therefore, the force detection model can be described as Equation (1).

(1) $$F_{pro}=F_{vis}+F_{con}+\overset{k_{1}}{\underset{i=1}{\sum }}F_{fri}+F_{sli}+\overset{k_{2}}{\underset{i=1}{\sum }}F_{def}$$

where $F_{pro}$ is the total proximal force; $F_{vis}$ is the natural viscous force; $F_{fri}$ is the combined friction force; $F_{sli}$ is the sliding friction; and $F_{def}$ describes the potential deformation energy.

## 4. Performance Evaluation Experiments and Results

This section describes the four types of experiment that were conducted to verify the performance of the developed ECRS. The purpose of experiment I was to establish a polynomial relationship between the magnetic field and the generated haptic force. Experiment II was used to evaluate the accuracy of the translation and rotation motion in the master haptic device. Experiment III was adopted to build the tracking performance. Lastly, we manipulated this ECRS to insert the guidewire and catheter into an EVE human model, described in experiment IV, which can clearly demonstrate the performance of the overall system.

### 4.1. Calibration Experiment of the Proposed Haptic Force Feedback

**Figure 11:**
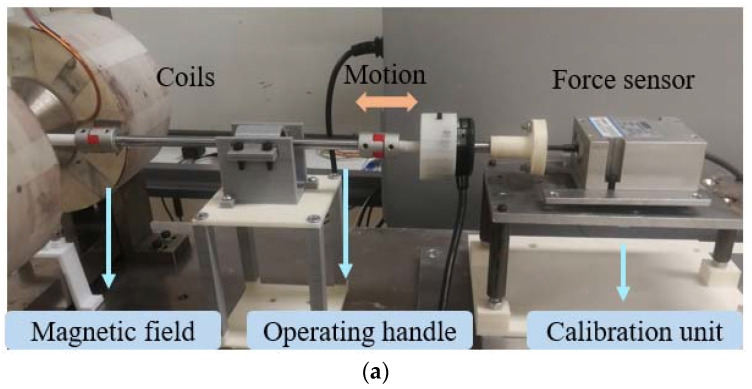

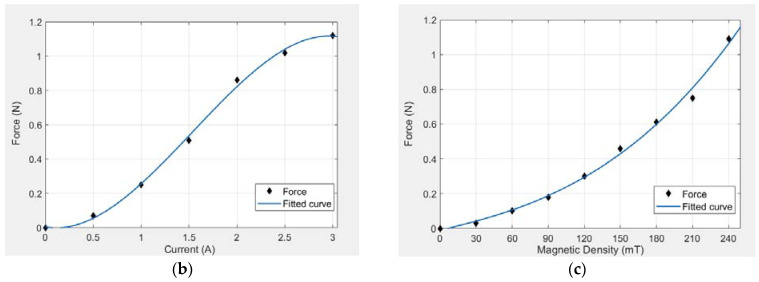
Calibration Experiment and results for the proposed haptic force feedback: (**a**) calibration experimental set-up; (**b**) relationship between supplied current and haptic force; (**c**) relationship between supplied magnetic density and haptic force.

In order to build the mathematical model between haptic force and the magnetic field, the experimental set-up was calibrated. The results for experiment I are displayed in Figure 11. We adopted a calibration unit based on a force sensor (Load Cell, Japan) to detect force information when powering the magnetic field. The operating handle was directly connected to calibration unit, and they were coaxial. At the beginning, we regulated the power supply to generate magnetic fields through the coils. Then, we slowly increased the thrust of the operating handle until relative sliding for the handle and force feedback structure occurred. Meanwhile, the calibration unit was able to capture the hitting force signal. In addition, we used two experimental methods to finish the calibration. The first one was to vary the supplied current strength to control the magnetic field within a fixed step of 0.5 A (the full range of the power supply is 3 A). The second method was to adjust the magnetic density from 0 mT to 240 mT at a fixed variation value of 30 mT. The value of the magnetic density was measured using a Tesla Meter (KANETEC, Japan). Note that a magnetic field density with a strong value was only used to calibrate the fitted relationship between the magnetic field and force in this part. A magnetic field over 200 mT is really not safe or stable. Generally, a secure laboratory magnetic environment is 100 mT to 130 mT. After fitting the experimental points using the cftool toolbox in MATLAB, it was possible to obtain two fitting third-order polynomials, which are shown as Equations (2) and (3).

(2) $$F_{i}=-0.0978i^{3}+0.45i^{2}-0.1025i+0.0052$$

(3) $$F_{mT}=\left(3.34e-08\right)m^{3}+\left(4.13e-06\right)m^{2}+0.0016m-0.0098$$

where $F_{i}$ represents the value of haptic force (current); *i* is the value of applied current; $F_{mT}$ is the value of haptic force (magnetic density); and *m* represents the value of magnetic density.

### 4.2. Accuracy Evaluation of the Master Operating Device

**Figure 12:**
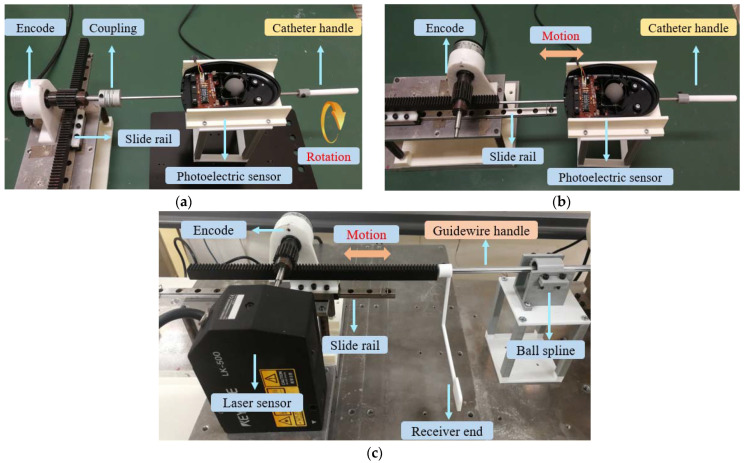
Accuracy evaluation experiments for the master operating device: (**a**) experimental setup for rotation evaluation of catheter operating handle; (**b**) experimental setup for translation evaluation of catheter operating handle; (**c**) experimental setup for linear motion evaluation of guidewire operating handle.

We organized three kinds of evaluation experiments to test the measurement accuracy of the proposed master operating device. The details of the experimental setup for translation and rotation are shown in Figure 12. The first two experiments were used to verify the detection accuracy of the photoelectric sensor (PS), which were mounted on the catheter operating handle. The purpose of third experiment was to evaluate the linear performance of the laser sensor (KEYENCE, Japan; the accuracy is 50 μm/mV), which was installed on the guidewire operating handle. During these three accuracy evaluation experiments, a sliding mechanical structured platform based on a rotating encode (MTL, MES020-2000P, Japan, the accuracy is 0.09 degrees/pulse) and slide rail (25 cm, Japan) was applied, to validate the measurement accuracy for both the operating handles.

In the rotation performance evaluation experiment (a), we used a rigid coupling to connect the catheter handle and the rotary axis of the sliding platform, to ensure absolute coaxial rotational motion. According to doctors’ natural surgical actions, the catheter operating handle was rotated using both counterclockwise and clockwise operations at a relatively gentle speed to reduce operation errors. We obtained the rotation accuracy by comparing the measured rotation angles of the encoder and the PS.

In the translation performance evaluation experiment (b), we placed the left end of catheter operating handle so that it directly contacted the linear rigid shaft with gears (the black shaft in Figure 12). It was necessary to ensure the isotropy of these two linear shafts. Both the inserting motion and the extracting motion were used to manipulate the catheter operating handle. By comparing the translation data detected by the PS and the encoder, the translation performance could be acquired.

In the accuracy evaluation experiment (c), the guidewire operating handle (GOH) and the black linear rigid shaft of the sliding platform were put on the same axis. The receiver end (white receiver piece) was printed with a polylactic acid (PLA) material to reflect the laser light emitted by the laser sensor. The purpose of this experiment was to test the linear-motion-detection accuracy of the guidewire operating handle using a laser sensor. The measurement data were processed using an Arduino Mega 2560 and monitored on a computer. It is worth explaining the reason for only conducting a linear accuracy experiment for the GOH; due to the guidewire handle also using an encoder sensor (see in Figure 2) to measure rotation information, using another encoder to test the accuracy would become meaningless. Therefore, we only conducted three related accuracy experiments in this part.

**Figure 13:**
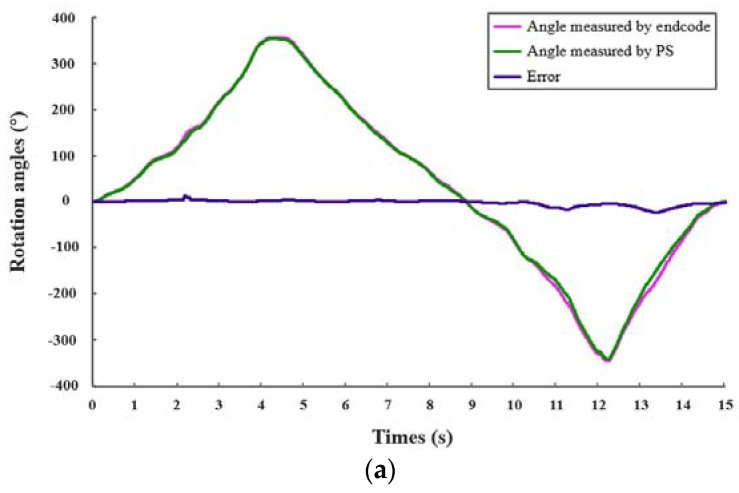

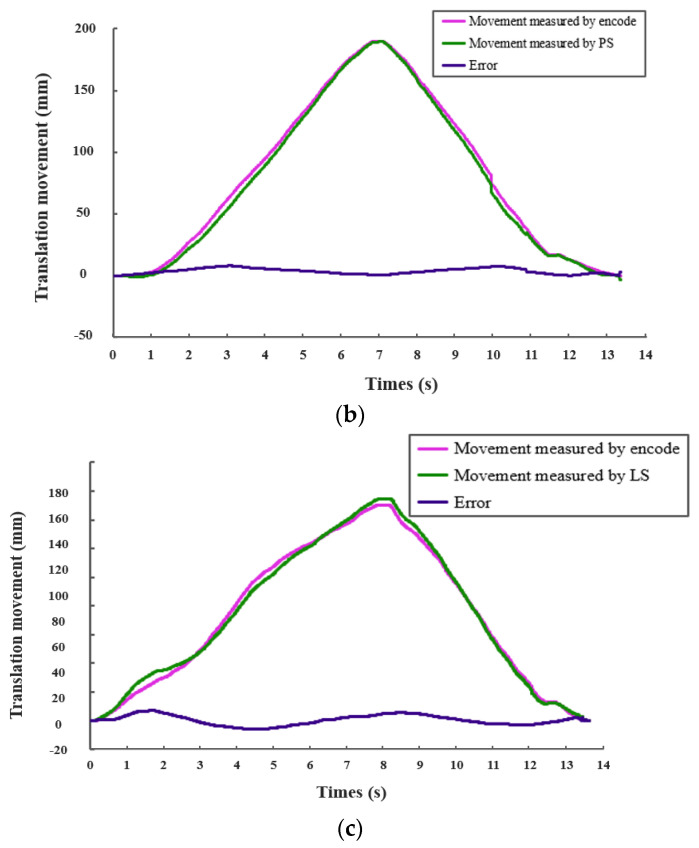
Experimental results for accuracy evaluation: (**a**) experimental result for rotation evaluation of catheter operating handle; (**b**) experimental result for translation evaluation of catheter operating handle; (**c**) experimental result for translation evaluation of guidewire operating handle.

The experimental results for the accuracy evaluation of the master operating handles are shown in Figure 13. Each kind of experiment was conducted five times. From the results of experiment (a) and experiment (b), some losses in coding steps can be. The reasons are unknown operational factors and non-absolute sliding of the trackball in the PS. The pink line is the angle or movement measured by the encoder. The green line is the angle or movement measured by the PS. The purple line is the error. In addition, the quantification of the measurement error is nonlinear. The value of average rotation moving error is 4.54%, and the value of average translation movement is 4.21%, which are satisfactory for surgeons to conduct surgeries using this catheter operating handle based on the PS.

The accuracy result for experiment (c) highlights a lot of trembling moving data points. This is because the designed receiver end is a 3D-printed PLA suspended structure instead of a rigid material. During the process of translation movement, the receiver end incurred a small range of front-to-back shaking. The pink line is the movement measured by the encoder; the green line is the movement measured by the LS; and the purple line is the error. After calculating the results in experiment (c), the minimum error is less than 0.2 mm, and the average translation error is 3.24 mm.

### 4.3. Tracking Performance of the Developed ECRS

In order to verify the tracking performance between master haptic device and slave manipulator-robot, we designed this part. By comparing the moving distance and rotation degrees detected on both the slave side and the master side, the tracking performance of this system was clearly demonstrated. In this experiment, we set the amount of movement in master device to 12 mm (corresponding to 1000 pulses for the encoder), and the theoretical movement value of the manipulation robot to 36.34 mm. For the rotation tracking performance in this part, we set 180 degrees as the input signal of the master device. The measurement rotation range of the slave side was 0 to 360 degrees. Each kind of experiment was conducted five times.

**Figure 14:**
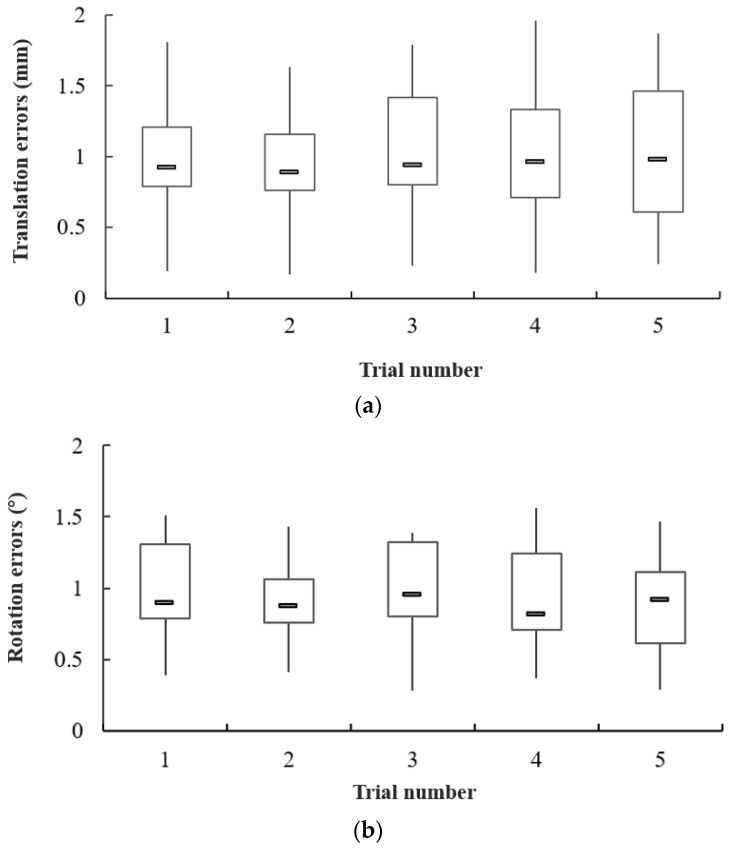
Experimental results for tracking performance between master side and slave side: (**a**) the result for translation over 5 trials; (**b**) the result for rotation over 5 trials.

The experimental results for tracking performance are shown in Figure 14. These data were collected by translation and rotation, respectively. From the box diagrams in Figure 14a, the average error of translation is 0.94 mm, the maximum error of translation is 1.96 mm, and the minimum error of translation is 0.17 mm. From the statistical result of rotation errors in Figure 14b, the average error of rotation is 0.89 degrees, the maximum error of rotation is 1.56 degrees, and the minimum error of rotation is less than 0.3 degrees. Based on the analysis of statistical tracking results, it is firmly believed that this developed ECRS is satisfactory for robot-assisted surgery.

### 4.4. Performance Evalution in In Vitro Experiments

**Figure 15:**
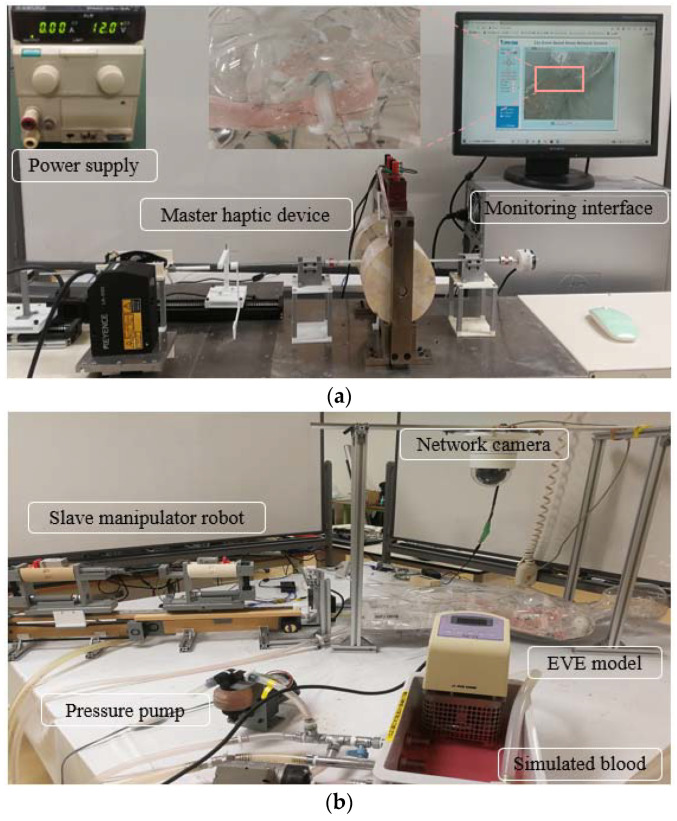
Experimental setup for in vitro experiments: (**a**) the view of master operation side; (**b**) the view of slave manipulator side.

To further verify the actual surgical performance of this developed ECRS, a trans-parent endovascular evaluator (EVE, Fain-Biomedical, Nagoya, Japan) human model was adopted in this experimental part. A pressure pump (KB-4N, Tokyo, Japan) was used to continuously inject simulated blood into the EVE human model, which is a very realistic surgical operating environment. In addition, we utilized a monitoring interface located on the slave side and displayed on the master side, to monitor the surgical status for insertion and extraction in real time. The details of the in vitro experimental setup are shown in Figure 15. Figure 15a is the designed master haptic operating side including two operating handles, a novel haptic feedback structure, a monitor interface, and a power supply. The slave manipulation side consisted of a slave manipulator-robot, an EVE human model, a press pump, and a network camera (VIV-TEK, San Jose, CA, USA). The camera had the ability to monitor the surgical status during the operating stage of experiment. Then, a real-time video was shown on the master side to guide the surgeries. Meanwhile, the operators could adjust the view of camera online at any time to ensure an excellent operational perspective. Regarding the introduction of surgical instruments, a J-type guidewire with a length of 260 cm and a catheter (Saitama, Japan) with a diameter of 5Fr (1Fr = 0.333mm) were used in this experiment.

**Figure 16:**
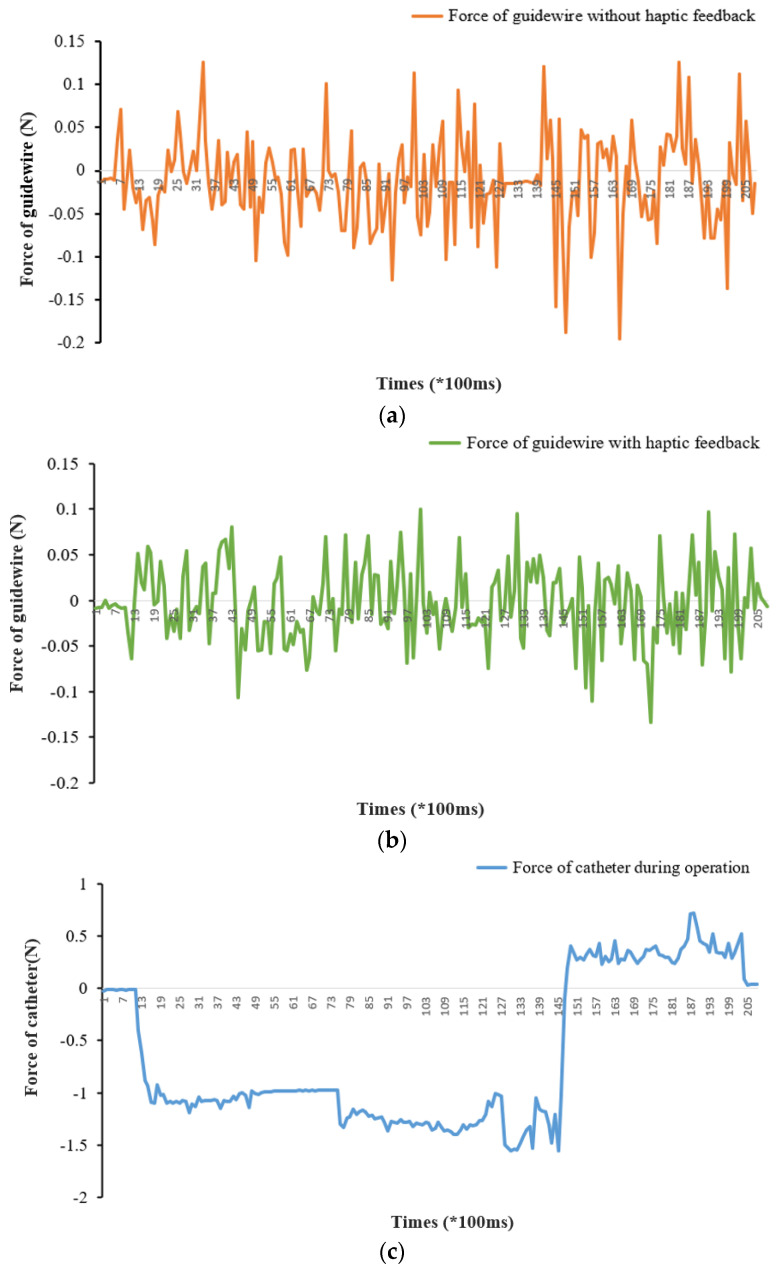
Experimental results for in vitro experiments: (**a**) the measured force of the guidewire without haptic feedback; (**b**) the measured force of the guidewire with haptic feedback; (**c**) the measured force of the catheter during the operation.

The in vitro experiment results are shown in Figure 16. We captured the real time force data using load cells and a related acquisition device. Figure 16a shows the variation in force when operating the guidewire into the EVE human model without haptic feedback. Figure 16b is the measured force information when operating the guidewire with the designed haptic feedback. Meanwhile, the change in force when operating the catheter into the Aortic arch of the EVE human model is shown in Figure 16c. It is worth mentioning that the feedback structure was mounted on the guidewire handle because the feedback experiments were only conducted on the movement of the guidewire.

**Figure 17:**
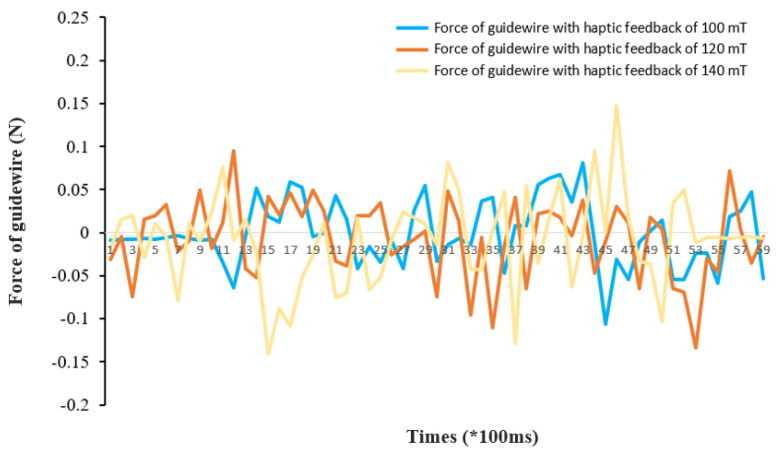
Quantitative experimental result with different levels of force feedback.

From the experiment results in Figure 16, it is very clear that force information measurements differ between operation with haptic feedback and without haptic feedback. The maximum force value is 0.195 N and the minimum force value is 0.0002 N in the experiment for the guidewire without haptic feedback (see Figure 16a). The maximum force value is 0.134 N and the minimum force value is 0.0007 N in the experiment for the guidewire with haptic feedback (see Figure 16b). Finally, the maximum force value is 0.195 N and the minimum force value is 1.553 N in the experiment for the catheter during the operation (see Figure 16c). Therefore, the study of haptic force feedback is vital for robot-assisted surgery, and can directly affect the force generated in the patient during surgery. Furthermore, we conducted three groups of guidewire force experiments with different levels of force feedback (100 mT, 120 mT, and 140 mT) to quantify the experimental result of force feedback. The quantitative experimental result with different levels of force feedback is depicted in Figure 17. As shown in the three differently colored data curves, the value of haptic feedback magnetic density can affect the stability and the maximum force value of the guidewire with haptic feedback. According to the literature [7], 110 mT is a stable reference magnetic field. The maximum force of the guidewire with a haptic feedback of 100 mT is 0.1067 N; the maximum force value with haptic feedback of 120 mT is 0.1338 N; and the maximum force value with haptic feedback of 140 mT is 0.14801 N. As shown in Figure 17, a force feedback environment of 100 mT and 120 mT is relatively safe and stable. The force information of the guidewire becomes a little bit abrupt when the magnetic density is 140 mT. The occurrence of this result might be due to the difficulty in precisely controlling the magnetic field.

**Figure 18:**
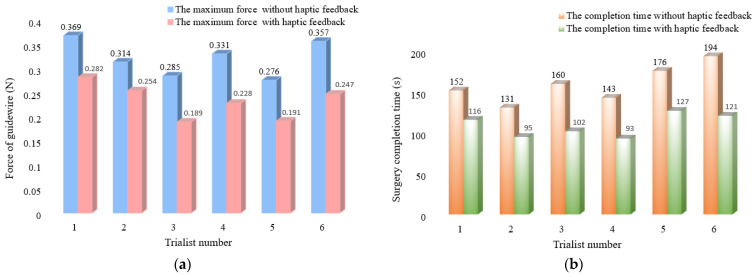
The statistical results for 6 trialists: (**a**) the maximum force value of guidewire without and with haptic feedback; (**b**) the surgery completion time for the operation task.

In addition, we invited six trialists to operate this developed ECRS in the in vitro experimental environment. At the beginning, training in the use of the operation method and attention paid to the items were necessary, since the trialists had different levels of medical operating knowledge. The force was measured using a load cell when the trialists operated this system on the EVE model. We counted the maximum force values in guidewire-inserting experiments of these six trialists; these values are shown in Figure 18a. Figure 18b displays the inserting-surgery completion time for all the participants.

From Figure 18a, we can see that the maximum force value with haptic feedback has a clear downward trend, and the average reduced force is 0.09 N. Such a reduction is significant in improving the safety of actual robot-assisted surgery. Figure 18b clearly displays that operations with haptic feedback are able to shorten surgical cycle times. The average reduced completion time is 50.33 s, which implies that physicians will be less burdened and more efficient in their surgical work.

## 5. Discussion

An ECRS is developed in this paper for robot-assisted vascular interventional surgery. This system makes it possible to follow doctors’ natural operating habits, and provides haptic feedback when conducting surgeries; it also has the potential application value of reducing workload, improving surgical environments, shortening surgery time, and enhancing operating safety for surgeons. Compared with the EC robots of other researchers, the proposed ECRS presents three significant contributions: Firstly, a cooperating master device based on two different independent handles is proposed, which can accurately drive the movement of the catheter and the motion of the guidewire; secondly, a unique structural design that can reduce operation tremors was adopted in the ECRS; and thirdly, a magnetically controlled haptic force feedback structure was designed based on hydrogels and magnetorheological (MR) fluids, which also has potential application value in the fields of haptic feedback, controlled clamping, and biological self-healing.

Regarding the tracking performance of this system, the average error of translation was 0.94 mm and the average error of rotation was 0.89 degrees. Despite our maintenance and cleaning before conducting the experiments, these errors still existed. This level of tracking error was generated by the unavoidable time loss in the slave manipulator and the precision deviation of mechanical manufacturing. Furthermore, due to the specialty of surgical instruments, the force information on the guidewire and catheter were distinctly different. The guidewire is hydrophilic, with easy bending of the head. Hence, the force of the guidewire was relatively small, usually less than 0.5 N. In contrast, the force of catheter was quite large due to the harder material, the friction between the guidewire and catheter, the interaction between the blood and the catheter body, and the contact between the vessel wall and the catheter. In particular, the catheter sheath for securing the surgical insertion position had a kind of necessary clamping force, which was a major influencing factor for the measurement of catheter force. Generally, the largest force value of the catheter was over 1.5 N. In addition, another influencing factor was whether or not trialists had operating experience. Skilled operation directly affected the smoothness of the manipulating procedure, surgery completion time, and the force data measured by the force sensor. Consequently, different types of subjects will be considered in the next robotic study and deep research.

## 6. Conclusions

In this paper, a developed endovascular catheterization robotic system (ECRS) with magnetically controlled haptic force feedback was presented, to complete collaborative operations and also to provide critical haptic feedback. Using the two operating handles to manipulate the catheter and guidewire, the ECRS is able to reproduce doctors’ natural skills. The ability to reduce operating tremors enables more accurate movement and motion data to be captured on the master side. Most importantly, we invented a magnetically controlled haptic structure based on solid MR and hydrogel, which can offer stable force feedback to operators. Our experimental results prove the feasibility of the developed ECRS, as well as its good performance in robot-assisted surgery.

Moreover, as one of potential advantages of the developed ECRS, the collaborative operation master device will have the effect of inspiring researchers to use master–slave robots in other fields, such as rehabilitation, ophthalmology, deep sea tasks, and air exit operations. Furthermore, the haptic force feedback with the function of magnetic control also has benefits in navigation, haptic perception, and controlled self-healing materials. In the future, research on multifunctional robotic systems and more accurate and smooth haptic force feedback will be considered and studied.

## Figures and Tables

**Table 1:** The related technical parameters of the designed master haptic device.

Items	Parameters	Values
System dimension (Maximum outer diameter)	Length	90 cm
	Width	26 cm
	Height	25 cm
Encode sensor	Shaft diameter	5.5 mm
Ball spline	Spline shaft length	15 cm
	Spline shaft diameter	6 mm
Laser sensor	Measuring range	30–50 cm
Guidewire operating handle (GOH)	Handle diameter	6 mm
Catheter operating handle (COH)	Handle diameter	6 mm
Screw	Effective distance	27 cm
